# Cognitive control and metacognitive awareness: do they shape academic achievement in university students?

**DOI:** 10.3389/fpsyg.2025.1633996

**Published:** 2025-10-10

**Authors:** Hande Musullulu, Javier Garcia-Orza, Diana Gómez Vázquez, Sara Garcia-Sanz

**Affiliations:** ^1^Department of Psychology, Saint Louis University Madrid, Madrid, Spain; ^2^Faculty of Education, Universidad del Atlántico Medio, Las Palmas de Gran Canaria, Spain; ^3^Faculty of Psychology and Speech Therapy, Universidad de Málaga, Málaga, Spain; ^4^Faculty of Education, Universidad San Pablo-CEU, CEU Universities, Madrid, Spain

**Keywords:** metacognition, metacognitive awareness, cognitive control, cognitive flexibility, academic achievement, inhibition, regulation of cognition, knowledge of cognition

## Abstract

Cognitive control and metacognition are important processes that significantly influence learning and academic achievement. Cognitive control facilitates goal-directed actions such as planning and monitoring, while metacognition enables individuals to effectively regulate their learning strategies. Although previous research has highlighted the importance of cognitive flexibility, inhibition and metacognitive regulation in academic performance, the interaction among these factors remains understudied. This study examined the relationship between cognitive control, metacognitive awareness (MA) and academic performance, as measured by grade point average (GPA), in university students. Two dimensions of cognitive control, cognitive flexibility and inhibition were measured using the Wisconsin Card Sorting Test (WCST) and the Go/No-Go Task, respectively. Metacognition was assessed through two subscales of the Metacognitive Awareness Inventory: knowledge of cognition and regulation of cognition. A hierarchical regression analysis revealed that cognitive flexibility was significantly related to GPA, with fewer perseverative errors on the WCST associated with higher GPA. Additionally, regulation of cognition, but not knowledge of cognition, explained further variance. Individuals who reported more frequent use of mental strategies to recognize and control their thinking had higher GPAs. Interestingly, a mediational analysis showed that metacognitive skills did not mediate the cognitive flexibility-GPA relationship. These findings emphasize the independent roles of cognitive flexibility and metacognitive regulation in influencing academic performance. Potential of training programs that target both cognitive control and metacognitive skills for the improvement of academic performance should be addressed in future studies.

## 1 Introduction

Academic success is a critical factor in determining the future prospects of university students, and understanding the factors that contribute to it can help improve educational outcomes and develop interventions to overcome the issues that it encompasses. A growing body of research has examined the relationship between cognitive control and academic achievement (Shi and Qu, 2021; Ganesan et al., 2024; Verma and Kar, 2024), while other studies demonstrated links between metacognition and academic achievement (Stanton et al., 2021; Bao et al., 2024). Interestingly, most of these studies have focused in school age children and not in university students, which is highly relevant as it seems that the relationship varies with age (Dvorak, 2024) and more importantly, most of these studies have investigated these factors in isolation, ignoring the relationship between cognitive control and metacognition (Cho et al., 2023; Swanson et al., 2024). Consequently, there continues to be a substantial gap regarding the complex dynamic interaction among these factors (Wu and Was, 2023), the current study seeks to clarify.

The present study aimed to contribute to the existing literature by exploring the relationship between, cognitive control (CC), metacognition and academic achievement through grade point average (GPA) among university students.

## 2 The definition of cognitive control

CC refers to a set of processes that enable goal-directed behavior through central mechanisms that modulate and guide other ongoing cognitive processes in order to flexibly adapt to changing environmental demands (Shepherd, 2023). CC is expressed in operations such as retrieving task-relevant information, suppression habitual responses, shifting between tasks and generating task rules (Friedman and Robbins, 2022). Miller and Cohen proposed a metaphor of CC, according to which the brain acts as “a set of tracks (*pathways*) connecting various origins (e.g., *stimuli*) to destinations (*responses*). The goal is to get the trains (*activity carrying information*) at each origin to their proper destination as efficiently as possible, avoiding any collisions” (Miller and Cohen, 2001, p. 184). This model posits that the prefrontal cortex (PFC), due to its anatomical connections with most cortical systems, as well as with the basal ganglia and limbic system, would be responsible for modifying the flow of information coming from other brain regions, inhibiting or enhancing certain processing pathways, in order to meet immediate demands and achieve specific goals.

An influential model suggested by Miyake et al. (2000), indicates that CC consists of three main components: *working memory, inhibition, and mental task shifting*. Apart from the traditional definitions of these constructs, they provide a broader definition of two of them. They describe working memory tasks as involving the updating and monitoring of relevant information. Mental task shifting on the other hand, encompasses several processes, including managing distractions, accessing necessary task information, and adapting to new tasks, essentially reflecting cognitive flexibility. Evidence from lesion studies and psychometric models shows that these three components are robustly correlated but separable when measured with latent variables (Friedman and Miyake, 2017). The common CC component, which is distinct from general intelligence, seems to be closely related to response inhibition, while working memory updating and mental set shifting appear to be cognitive processes distinct and separable from common CC (Friedman and Robbins, 2022).

Although CC is often used interchangeably with executive functions (EF), they are not conceptually identical. CC primarily refers to the capacity to regulate mental processes and efficiently managing information such as maintaining and updating representations, inhibiting automatic responses and flexibly adjusting behavior and information flow to meet contextual demands (Friedman and Miyake, 2017). Thus, it enables individuals to adapt and exert control over their cognitive operation (Alzahabi et al., 2022). EF, on the other hand, are described as the capacity to synchronize cognitive processes and behavioral responses in the pursuit of goal-directed activity (Pluck et al., 2023). For instance, CC focuses on how mind regulates behavior to reach goals, whereas EF as a broader set of higher-order functions involving working memory, inhibition, activation selection and episodic retrieval (Badre, 2025). Overall, although CC and EF are closely related and often co-activated, CC focuses on internal cognitive regulation, while EF extends this regulation to overt behavior and complex decision-making.

### 2.1 Cognitive control and academic achievement

Within the framework of CC, cognitive flexibility and inhibition are two crucial components which could have a role in academic performance. Cognitive flexibility is the ability to adapt to changes, perform task-switching and adaptation of cognitive strategies in novel or unexpected contexts (Uddin, 2021). In the academic context, cognitive flexibility aids in adapting to various learning concepts, employing different strategies based on a specific task or question and easily switching between tasks (Zheng et al., 2024). Studies indicate that cognitive flexibility is positively associated with academic outcomes such as reading, mathematics and overall academic performance (Huang et al., 2022; Gökçe and Güner, 2024).

On the other hand, inhibition refers to the ability to actively suppress dominant or automatic responses and to resist interference from external stimuli, in order to present goal-directed behavior. It involves active self-regulation toward impulses pause and proactive control (van den Wildenberg et al., 2022; Werner et al., 2022). One study has shown that inhibition, as measured by the Stroop test, predicts mathematics ability in college students (Coulanges et al., 2021). Rea-Sandin et al. (2021) further support this by showing the relationship between educational attainment and inhibitory control in children with an average age of 9 years.

Recognition of these two components of CC is crucial, as they influence academic performance and support academic achievement. Importantly, one study of university students found a weak correlation between inhibitory control, as measured by the Stroop Test, and academic performance, suggesting that the relationship may be more complex or context-dependent (Dvorak, 2024). Similarly, because academic achievement is multifaceted, cognitive flexibility alone is also not a reliable predictor. In order to gain a more comprehensive understanding of how academic success develops, it is necessary to consider the impact of these two factors and explore other additional ones (Nakhostin-Khayyat et al., 2024).

## 3 Defining metacognition

As first proposed by Flavell (1979), metacognition is a form of cognitive modeling that involves higher mental processing, described as “*thinking about thinking*.” Processes such as mental flexibility, judgment ability, monitoring, decision making, self-regulation, and other skills are implicated in metacognition. Metacognition allows individuals to gain awareness regarding what they learn, know, and think, as well as the ability to regulate themselves and their activities in a constructive way. Individuals with high-level metacognitive abilities generally derive positive experiences from their daily lives using effective attentional and self-regulatory strategies and constructive practices (Stanton et al., 2021). In contrast, low metacognitive skills are commonly associated with high rates of psychopathology and negative outcomes across a wide range of life domains.

Based on previous seminal theories (Flavell, 1979; Schraw and Dennison, 1994), there are two proposed two subcomponents of metacognition: knowledge of cognition and regulation of cognitions. Knowledge dimension involves aspects related to declarative, procedural and conditional knowledge. Regulation dimension cover planning, information management strategies, monitoring, debugging strategies and evaluation. In other words, it is the ability to control learning related activities such as thinking (Stanton et al., 2021). Metacognitive knowledge, otherwise known as metacognitive awareness (MA), entails what the learner knows about their own cognitive processes, such as factors that may interfere with learning, knowing the appropriate timing of information use, and learning strategies. Furthermore, knowledge of cognition is a broad term involving three subdivisions. Firstly, declarative knowledge includes the knowledge the learner has regarding themselves and the factors that may hinder their performance. Secondly, procedural knowledge involves all types of strategies the learner knows about during a task execution. Lastly, conditional knowledge refers to knowing when and how to apply these strategies.

Several other traditional frameworks have also shaped the contemporary understanding of metacognition. One of these is the two-level model, which was developed by Nelson and Narrens (1990). This model presents two interrelated levels of cognitive processes named the object level and the meta level. The object level involves task-primary level operations such as information processing, decoding and comprehension, while the meta level constitutes a higher-order system responsible for monitoring and controlling the goals set by the learner. The meta level monitors and regulates the information in the object level. This bidirectional flow enables information to be transferred from the object level to the meta level through monitoring, and back from the meta level to the object level through control. In other words, the meta-level enables the learner to judge if the learning outcomes are satisfactory or requires strategy adjustment. However, the interactive model developed by Efklides (2011, 2014)) proposes that metacognition comprises three-layered interactive model. These layers include metacognitive knowledge (beliefs about cognition), skills (control strategies), and experiences (emotions and judgments). The model suggests that these levels can be categorized as follows: the “object level” (unconscious and automatically regulated), the “personal awareness level” (conscious and intentionally controlled), and the “social awareness level” (monitoring and reflecting others’ understanding). Therefore, the model advances the argument that metacognition is not only cognitive but also experiential, motivational, affective, and occasionally social (Efklides, 2014).

A more recent theory was proposed by Lebuda and Benedek (2023) called Creative Metacognition Framework (CMC) building on earlier models by distinguishing between core cognitive and metacognitive processes. Another theory that takes a more distinct approach is the “model-free” metacognition theory proposed by Carruthers and Williams (2022), constituting a more ancestral and elementary internal alert system, thus eliminating the necessity for such complex mental processes. Direct signals are used to represent a mental state or process, automatically guiding one’s actions without the requirement for the subject to “think about their thinking.” In a mouse navigating a complex maze, hesitation signals cognitive effort without conscious evaluation in animal models. Similarly, individuals may pause a difficult task without consciously monitoring their performance. This reflects an internal signal of cognitive effort rather than a conscious evaluation of memory or attention. Together, all these frameworks emphasize both the monitoring and regulatory dimensions of metacognition as well as their social, adaptive and developmental factors behind it.

### 3.1 Metacognition and academic achievement

Effective learning is a critical factor for academic success, requiring the development and application of metacognitive skills (Fleur et al., 2021). Existing research has frequently underlined the contribution of metacognitive skills to both learning and academic achievement (Santangelo et al., 2021). Tuononen et al. (2023) noted that MA, specifically knowledge and regulation of cognition, predicted organization skills and use of a deep approach while studying. Another study reported a positive correlation MA and academic outcomes among students (Agrawal et al., 2025). On the other hand, Deshmukh et al. (2025) reported similar results only when intrinsic motivation was also present. Similar results were observed in another study showing that both metacognition and “need for cognition,” that is closely tied to motivation and reflective thinking, were strong predicters of academic success (measured by GPA) among university students (Özçakmak, 2021). On the contrary, another study found no relationship between metacognition and academic success measured with GPA, arguing that the results could be attributed to environmental factors, motivation or self-efficacy (Mackewn et al., 2022). Nevertheless, Pucillo and Perez (2023) unveiled that MA could explain 31% of the variation in GPA among university students. These collective findings emphasize the role of metacognitive skills and provide support for the idea that the effective use of metacognitive skills, especially MA, positively impact learning outcomes and academic achievement.

## 4 The current study

Given the wide range of processes involved, metacognition is of immense importance in the field of education, both in theoretical and practical terms. By allowing individuals to self-organize and effectively manage high cognitive processes, it contributes significantly to academic self-confidence, time planning skills and the ability to find appropriate strategies for themselves (Fleur et al., 2021). While CC is implied in planning, monitoring, adjustment and other similar executive processes, metacognition is considered to play a role in controlling activity and applying optimal strategies for different conditions.

Drawing on the perspectives outlined above, this study proposes that CC and metacognition should not be viewed as separate predictors of academic achievement. Instead, their interaction may be interdependent or play distinct roles in shaping academic outcomes. To explore this, the study addresses two key research questions. (1) Are the dimensions of metacognition, namely knowledge of cognition and regulation of cognition, related to CC components and GPA? (2) If such relationship exists, do these metacognitive components fully or partially mediate the link between CC and GPA?

## 5 Methods

### 5.1 Research protocol and ethical considerations

The research protocol was previously registered in the Open Science Framework (OSF) where the research data were also stored with the following link: https://osf.io/jv67y/ (last accessed February 7, 2025). The data were collected by three researchers between April and June 2024.

This research followed the Declaration of Helsinki and was approved by the ethical committee of Universidad del Atlántico Medio (CEI/02-10) on February 26, 2024. The entire procedure was conducted according to ethical principles of the European Code of Conduct for Research Integrity (ALLEA, 2023). It was ensured that all data collection was conducted anonymously, with no personal identity information gathered from participants. Prior to the experiments, participants were informed of their rights and provided with a detailed description of the research through informed consent, in which their written consent was obtained. While participation was voluntary and entailed no financial compensation, as a non-monetary incentive, students received points toward the participation component of their course grade (accounts for 5% of the total grade) for taking part in the study.

### 5.2 Participants

The sample primarily comprised of 91 students from a private university in Gran Canaria, Spain. One participant was removed for not completing one of the questionnaires, and three additional participants were excluded due to outlier values exceeding three standard deviations on at least one measure. The final sample included 87 undergraduate Psychology students (*N* = 87, 83.9% female). The age ranged from 18 to 42 (*M* = 19.9, SD = 2.9).

The participants were invited to participate regardless of age and gender. All Spanish-speaking students from the undergraduate psychology course were included in the study to ensure a comprehensive representation of the student population. Likewise, no stipulated minimum GPA value was introduced as an eligibility criterion for participation as it was solely used as a measure for academic achievement.

### 5.3 Piloting and data collection procedure

A pilot study involving four students was conducted to prevent any misapprehension of the questions or the tasks which may arise during the data collection procedure. Participants were asked to provide feedback regarding the computerized tasks, the clarity of the questionnaires and whether they experimented fatigue during the sessions. Based on the feedback received, adjustments were made for the testing procedure.

Data were collected in a multimedia room equipped with computers. All the data collection procedure was conducted in this room to ensure a consistent environment for all the participants. Psytoolkit (psytoolkit.org; Stoet, 2010, 2017), an online platform for data collection, was used during the experimental procedure. Per each data collection session, which approximately took 20 min, a maximum of 10 participants were admitted ensuring a standardized environment. Each participant was assigned an individual computer to complete the testing under identical conditions. Upon arrival, all participants were provided with a standardized briefing on the study stages and the computerized tasks they would complete in addition to on-screen instructions for each task.

### 5.4 Measures

Prior to completing the computerized tasks, participants filled out a brief survey detailing demographic information, including age, gender, and highest education degree completed. During this survey, two other pieces of information were required. *Primarily*, the participants were requested to disclose their GPA (with a maximum mean score of 10.00) from the previous semester, a detail subsequently verified by a researcher during the experiment to ensure the reliability and accuracy of the participant’s response. Moreover, the GPA was deemed of interest to employ as a measure for academic achievement. Given the potential variability in GPA between faculties, owing to differences in course demands specific to each faculty’s curriculum, the sample was restricted to participants from the psychology major only.

After the completion of the brief sociodemographic questionnaire which involved along with other questions indicated previously, enclosing academic achievement through GPA, CC and metacognition were assessed through neurocognitive tests. CC was measured by means of computerized tasks of cognitive flexibility (Wisconsin Card Sorting Test) and inhibition (Go-No Go Task) suggested by Shepherd (2023). Metacognition was measured by using Metacognitive Awareness Inventory by Schraw and Dennison (1994) which contains two scales: knowledge of cognition and regulation of cognition. The order of the computerized tasks was randomized to reduce potential bias which may arise due to the sequencing of the tasks.

### 5.5 The Go/No-Go Task

The Go/No-Go Task is an experimental paradigm utilized to measure inhibition and impulsivity responses (Donders, 1969; Montalti et al., 2024). The task was conducted using Psytoolkit^1^ with the task structure kept unchanged. Task instructions were provided on screen before the task commenced.

The task included two stimuli, green “go” signal and red “no-go” signal within circles. With each “go” signal, participants were required to press the space bar within 2 s. For “no-go” signals, they had to withhold themselves from pressing the space bar. Participants were displayed feedback indicating delayed response if they responded late, and error, if they responded to the no-go signal.

Commission errors (pressing the button during no-go trials) and omission errors (missing go trials) were recorded although only the former were measured as a measure of inhibition. Every participant received a total of 25 trials, consisting of 5 “no-go” commands and 20 “go” commands.

### 5.6 The Wisconsin Card Sorting Test (WCST; Grant and Berg, 1948)

To measure cognitive flexibility, computerized version of the Wisconsin Card Sorting Test (WCST) was used. The original WCST involves a larger set of stimulus cards, however for this study the most recent version, WCST-64 (Greve, 2001), was chosen and conducted through Psytoolkit.^2^ This experimental paradigm involves presenting participants with 64 stimulus cards, each requiring classification among four stacks. The classification of the card varies based on its shape, color or quantity with the rule changing every 10 cards, necessitating participants to adapt to novel rule. The reason WCST was employed was due to its capacity to measure cognitive flexibility through task switching and inhibition by introducing an aspect on suppressing responses which do not comply with the context or rules utilized to assess cognitive flexibility (Miyake et al., 2000; Miles et al., 2021). Although WCST is a widely used neurocognitive task to assess cognitive flexibility, some concerns have been raised regarding the lack of consensus on scoring methods which may affect the interpretation of results (Miles et al., 2021).

During the testing, participants received feedback each time they did a classification indicating their response as “correct” or “incorrect” which served them as a guide to change their strategy and adapt to the shifting rules. Perseverative errors, all consecutive errors after the ending of the second trial in the series were collected and constituted our dependent variable. Slow responses (response time > 10 s) were equally considered perseverative errors if these followed a previous incorrect response.

### 5.7 Metacognitive Awareness Inventory (MAI, Schraw and Dennison, 1994)—Spanish version (MAI-SP, González-Cabañes et al., 2022)

The assessment of metacognition was carried out by using the Metacognitive Awareness Inventory (MAI) developed by Schraw and Dennison (1994) a well-known and commonly used scale for measuring metacognitive abilities. The MAI has been validated and widely used in educational psychology research, making it a robust tool for assessing metacognitive processes. It is a self-report questionnaire involving 52 items based on a true/false scoring system, consisting of two dimensions: knowledge about cognition and regulation of cognition.

Knowledge about cognition includes three subcategories: (a) Declarative knowledge, which refers to the capacity to understand one’s own abilities and intellectual resources; (b) Procedural knowledge, which involves knowing how and when to use strategies; and (c) Conditional knowledge, which implies the ability to know why and in which contexts these strategies should be used. The distribution of the items for each category is as follows: declarative (8 items), procedural (4 items), and conditional knowledge (5 items).

Regulation of cognition encompasses five subcategories: (a) Planning, which involves setting goals and allocating resources before learning activities; (b) Information management strategies, which include organizing and processing information efficiently; (c) Comprehension monitoring, which refers to the ongoing assessment of one’s understanding during the learning process; (d) Debugging strategies, employed to identify and correct comprehension and performance errors; and (e) Evaluation, which entails analyzing the effectiveness of learning strategies and performance post-learning. The distribution of items for each category is as follows: planning (7 items), information management strategies (10 items), comprehension monitoring (7 items), debugging strategies (5 items), and evaluation (6 items).

Harrison and Vallin (2018) proposed an adapted version of this scale which included an updated response scale to reduce acquiescence bias (Likert type scale from 5 = “very typical of me” to 1 = “not at all typical of me”). They also found that the 52 items version had poor fit and that a short version with 19 items and the same two original dimensions showed better statistical properties. Since the original questionnaire is published in English, the validated version in Spanish of MAI (MAI-SP), developed by González-Cabañes et al. (2022) based on the Harrison and Vallin’s version was employed for the current study. The MAI-SP included both the short and long version of the questionnaire. Aligned with the results of the Harrison and Vallin’s study (2018), the short version showed better fit and demonstrated good internal consistency, with Cronbach’s alpha values of 0.812 for knowledge of cognition and 0.772 for regulation of cognition. Moreover, strong correlations for test–retest reliability were reported. Despite this, we applied the long version (that also includes the short version) in the current study to enhance the comprehensiveness of the questionnaire and address a gap in the literature, as the long version has not been previously used in Spanish population. Before its application, the MAI-SP was revised again by two native psychologists and one expert linguist, for ensuring the cultural appropriateness of the scale for the population of Gran Canaria as the original work done by González-Cabañes et al. (2022) was conducted in the Iberian Peninsula. During this revision phase, interrater agreement between these 3 professionals was established regarding the clearness and conciseness of the statements. Following this phase, no changes were made.

## 6 Results

The analyses were conducted using IBM SPSS Statistics v. 28. Table 1 displays the descriptive statistics for academic achievement, measured as GPA, and the two cognitive control tasks. Inhibition was assessed by the number of commission errors in the Go-No-Go Task, while cognitive flexibility was measured by the number of perseverative errors in the WCST. The table also includes the scores for the subscales of the MAI, specifically, knowledge of cognition and regulation of cognition. These subscales were examined according to the structure proposed by Schraw and Dennison (1994) which uses 52 items, as well as the reduced 19-item version identified by González-Cabañes et al. (2022) and (Harrison and Vallin, 2018).

**Table 1:** Descriptives for the sample in academic achievement, cognitive control tasks and subscales of the MAI both in the long (52 items) and short versions (19 items).

Variable	Minimum	Maximum	Mean	Standard error	Standard deviation
GPA	5.00	9.45	7.45	0.11	1.05
WCST-Perseverations	4	17	7.45	0.28	2.59
Go/No-Go-Commissions	0	2	0.35	0.06	0.56
MAI KC-52	2.50	4.72	3.63	0.06	0.56
MAI RC-52	2.60	4.66	3.63	0.05	0.47
MAI KC-19	1.88	4.75	3.45	0.07	0.66
MAI RC-19	2.55	5.00	3.64	0.06	0.56

GPA, Grade Point Average; WCST-Perseverations, Number of perseverations in the Wisconsin Card Sorting Test; Go/No-Go-Commissions, Number of commission errors in the Go/No-Go Task out of 5 items; MAI KC-52, knowledge of cognition subscale of Metacognitive Awareness Inventory long version; MAI RC-52, regulation of cognition subscale of Metacognitive Awareness Inventory long version; MAI KC-19, knowledge of cognition subscale of Metacognitive Awareness Inventory short version; MAI RC-19, regulation of cognition subscale of Metacognitive Awareness Inventory short version.

Before proceeding with further analyses, the internal consistency of the MAI was evaluated. For the 52-item version, Cronbach’s alpha was 0.87 for knowledge of cognition and 0.90 for regulation of cognition. The reduced 19-item version showed slightly lower values, with a Cronbach’s alpha of 0.81 for knowledge of cognition and 0.78 for regulation of cognition. These findings align closely with those reported by González-Cabañes et al. (2022) who found values of 0.81 and 0.77 for the respective subscales. These results confirm the internal consistency of both the full and reduced versions of the MAI (MAI-SP).

Table 2 presents the Spearman correlations between the study’s key variables. GPA showed non-significant inverse relationship with the number of perseverative errors in the WCST and with the number of commission errors in the Go-No-Go Task. However, GPA was positively correlated with both the long and short versions of the knowledge of cognition and regulation of cognition subscales. Among the cognitive control measures, the Go-No-Go Task exhibited no significant correlations with any other variables, likely due to floor effects. In contrast, the number of perseverative errors in the WCST, a measure of cognitive flexibility, was significantly correlated with the knowledge of cognition subscale in its short form and marginally in its long form (*p* = 0.057), but not with the regulation of cognition subscale. As anticipated, the metacognitive subscales displayed moderate to high intercorrelations, with all values exceeding rs = 0.69 (*p* < 0.001). These findings support the expected relationships between metacognitive components.

**Table 2:** Spearman’s correlations among academic achievement score, cognitive control tasks and subscales of the MAI both in the long (52 items) and short versions (19 items).

	GPA	WCST-perseverations	Go/No-Go-commissions	MAI KC-52	MAI RC-52	MAI KC-19	MAI RC-19
GPA	–						
WCST-Perseverations	−0.16	–					
Go/No-Go-Commissions	−0.06	−0.01	–				
MAI KC-52	0.45^**^	−0.21	0.04	–			
MAI RC-52	0.41^**^	−0.14	0.00	0.80^**^	–		
MAI KC-19	0.42^**^	−0.25^*^	0.08	0.92^**^	0.72^**^	–	
MAI RC-19	0.46^**^	−0.08	−0.02	0.77^**^	0.94^**^	0.69^**^	–

Significance levels (bilateral): *p < 0.05, **p < 0.01. GPA, Grade Point Average; WCST-Perseverations, Number of perseverations in the Wisconsin Card Sorting Test; Go/No-Go-Commissions, Number of commission errors in the Go/No-Go Task out of 5 items; MAI KC-52, knowledge of cognition subscale of Metacognitive Awareness Inventory long version; MAI RC-52, regulation of cognition subscale of Metacognitive Awareness Inventory long version; MAI KC-19, knowledge of cognition subscale of Metacognitive Awareness Inventory short version; MAI RC-19, regulation of cognition subscale of Metacognitive Awareness Inventory short version.

### 6.1 Hierarchical regression analysis

The primary aim of this study was to examine whether and how CC (measured by cognitive flexibility and inhibitory control) and metacognitive skills are related to academic achievement in university students. To address this, we conducted least squares hierarchical regression analyses followed by a mediation model. Due to floor effects and minimal variability in the inhibitory control task (i.e., the Go/No-Go Task), this variable was excluded from the analyses. Consequently, only the number of perseverative errors, a measure of cognitive flexibility, was included as a cognitive control variable.

For the metacognitive variables, we selected the knowledge of cognition and regulation of cognition subscales from the shortened 19-item version of the MAI because of its superior psychometric properties, including better internal consistency and more robust factor structure. As indicated previously, a confirmatory factor analysis conducted by González-Cabañes et al. (2022) supports the two-factor model using this shortened version, as it provided the best fit among competing models, including the one-factor, two-factor, and eight-factor structures derived from the full 52-item version, as well as the unifactorial model using the 19-item version. Their findings align with those reported for the English adaptation of the instrument (Harrison and Vallin, 2018).

GPA was the dependent variable, and cognitive flexibility was entered as the first predictor in the model. To investigate whether metacognitive factors account for additional variance beyond cognitive flexibility, the knowledge of cognition and regulation of cognition subscale scores from the shortened version of the MAI were entered in the second step.

Visual inspection of the residuals indicated a normal distribution. Multicollinearity was assessed using variance inflation factors (VIF), with all values below 1.1 and tolerance values above 4.5, confirming no issues with collinearity.

The results of the hierarchical regression analysis are presented in Table 3. Cognitive flexibility accounted for 6.6% of the variance (adjusted *R*^2^ = 5.4%) in GPA, *F*(1, 84) = 5.89, *p* < 0.017, with students displaying more perseverative errors having lower GPA scores. When the two metacognitive variables were entered in the second step, there was a significant change *R*^2^, Δ*R*^2^ = 17.3, *F*(2, 82) = 9.33, *p* < 0.001, increasing the total variance explained to 23.9% (adjusted *R*^2^ = 21.1%). Cognitive flexibility remained a significant predictor (*p* = 0.032). Higher scores in regulation of cognition were significantly associated with higher academic achievement (*p* = 0.005), while knowledge of cognition did not significantly predict GPA (*p* = 0.843).

**Table 3:** Hierarchical regressions with cognitive flexibility (WCST-Perseverations) and metacognitive skills (knowledge of cognition and regulation of cognition subscales of the short version of MAI) as predictors of GPA.

Predictors	*b*	*SE b*	*β*	*t*
Step 1				
WCST-Perseverations	−0.104	0.043	−0.256	−2.427*
*F*(1, 84) = 5.89*, *R*^2^ = 0.066				
Step 2				
WCST-Perseverations	−0.091	0.042	−0.224	−2.181*
MAI KC-19	0.046	0.230	0.029	0.199
MAI RC-19	0.742	0.257	0.397	2.886**
*F*(3, 82) = 8.572**, *R*^2^ = 0.239				

Significance levels (bilateral): *p < 0.05, **p < 0.01. GPA, Grade Point Average; WCST-Perseverations, Number of perseverations in the Wisconsin Card Sorting Test; MAI KC-19, knowledge of cognition subscale of Metacognitive Awareness Inventory short version; MAI RC-19, regulation of cognition subscale of Metacognitive Awareness Inventory short version.

### 6.2 Mediation analysis

The regression analysis indicated a relationship between cognitive flexibility, metacognitive skills, specifically Regulation of Cognition, and academic achievement. To explore *how* these variables are related, we conducted a mediation analysis. From a theoretical standpoint, we hypothesized that better regulatory skills would allow students to better leverage executive functions, thus enhancing GPA.

We conducted the mediation analysis using ordinary least squares path analysis. The number of perseverations in the WCST was included as the independent variable, while the two reduced versions of the MAI subscales, regulation of cognition and knowledge of cognition, served as mediators, with GPA as the dependent variable. The PROCESS macro for SPSS (Preacher and Hayes, 2008, Model 4) was used to test the mediation model.

**Figure 1:**
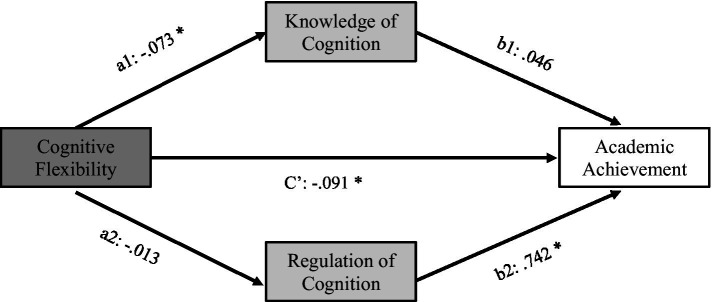
Unstandardized coefficients of the mediation model. Total effect of cognitive flexibility on academic achievement: *c* = −0.104, *t* = −2.42, *p* = 0.0173, CI [−0.189, −0.018]; indirect effects of cognitive flexibility on academic achievement mediated by knowledge of cognition: a_1_b_1_ = −0.003, CI [−0.039, 0.032] and by regulation of cognition: a_2_b_2_ = −0.009, CI [−0.047, 0.020]. *p < 0.05.

Figure 1 displays the results of the analysis, indicating a complex relationship between the factors. A direct effect of cognitive flexibility on academic achievement was found. Cognitive flexibility was also related to knowledge of cognition, but this factor did not directly affect academic achievement. Regulation of Cognition was not related to cognitive flexibility, but it was significantly related to academic achievement.

To examine the indirect effects of the mediators on the relationship between cognitive flexibility and academic achievement, we used bootstrap confidence intervals with 5,000 samples. Neither knowledge of cognition (a_1_b_1_ = −0.003, CI [−0.039, 0.032]) nor regulation of cognition (a_2_b_2_ = −0.009, CI [−0.047, 0.020]) showed intervals excluding zero, indicating that the metacognitive variables did not mediate the relationship between cognitive flexibility and academic achievement.

## 7 Discussion

This study addressed two main research questions. The results indicate a complex interplay between cognitive flexibility, metacognition, and academic achievement through GPA, summarized as follows.

Regarding the first research question, cognitive flexibility was directly related to both knowledge of cognition and academic achievement. While dimensions of MAI also correlated with GPA, hierarchical regression analysis revealed that, after accounting for cognitive flexibility, only regulation of cognition remained a significant predictor of academic achievement. These results are aligned with the conceptualizations of Nelson and Narrens (1990) and Efklides (2014). Regulation of cognition involves active monitoring, use of strategies and other control decisions during learning but also metacognitive experiences which involve feelings, judgments about understanding, self-efficacy and confidence (Efklides and Schwartz, 2024). For example, learners with higher regulation of cognition may pause and re-read the same passage to improve comprehension, or they may change their approach or implement new strategies when encountering difficulties during overall learning-related tasks.

Knowledge of cognition was not a significant predictor of GPA once regulation of cognition was included. This demonstrates that knowledge of cognition could be insufficient to predict GPA alone, as one’s awareness of their own strategies may not always guarantee their implementation. This pattern also highlights an important distinction between metacognitive knowledge and regulation, revealing that there could be a potential gap between knowledge and skills layer of metacognition (Efklides, 2014). Furthermore, motivation and self-regulation have been identified as factors that influence knowledge of regulation (Acosta-Gonzaga and Ramirez-Arellano, 2021) which could explain the pattern of current findings.

Concerning the second question, mediation analysis did not support a mediation model. Instead, cognitive flexibility and regulation of cognition independently contributed to academic achievement, highlighting their distinct roles in influencing GPA as previously supported (Gökçe and Güner, 2024; Deshmukh et al., 2025). The underlying reasons for the independent impact of these two variables on academic achievement may be attributable to the notion that cognitive flexibility constitutes a mental capacity that fosters adaptive thinking, while regulation of cognition is derived from strategically and actively controlling learning processes. The finding that both factors are multifaceted suggests that they may have influenced academic achievement through different pathways. Put differently, the absence of mediation may indicate that high cognitive flexibility does not automatically translate into high regulation of cognition. Conversely, strong regulation of cognition may not necessarily imply greater cognitive flexibility, highlighting their independent contributions to academic achievement. Our findings are parallel to prior research that found no correlation between combined influence of multiple EF domains and metacognitive knowledge monitoring (Wu and Was, 2023).

Despite the correlation between some of these measures, by showing independent contributions of regulation of cognition and cognitive flexibility, we add to previous findings that support the multifaceted character of academic performance. Collectively, these findings also support the results from previous findings that studied the individual contribution of these factors to academic achievement (Nugiel et al., 2023; Pucillo and Perez, 2023). Hence, it is important to note that the findings of the current study contribute to a clear focus on CC through cognitive flexibility, rather than testing broad EF functions. Moreover, it showed the impact of two different metacognitive dimensions and employed testing mediation with different methods.

It is noteworthy to highlight that inhibition was not found to be correlated with metacognition or academic achievement. Previous studies have shown the important role of inhibition in academic success from early childhood to adulthood (Privitera et al., 2023). One study conducted among 107 university students demonstrated that students with lower academic performance made more errors on the Stroop Test, which is an instrument widely use to assess inhibition (Dvorak, 2024). This may be due to the methodological aspects behind the experimental paradigm employed in the current study.

Previous studies have shown that academic performance increases with MA, highlighting its role in educational achievement (Summiya, 2024). Individuals who have a high level of awareness of their metacognitive processes may have positive academic performance because they are able to identify their learning strategies and have good self-management skills. Dangin et al. (2023) revealed that MA and self-regulated learning predict academic performance, explaining 24.8% of student GPA variance. Another study found negative correlation between the MAI and academic performance measured by end of year assessment scores (−0.029) among students. This may be attributed to potential illusory bias when judging metacognitive abilities and academic achievement (Hassan et al., 2022).

The results of the current study showed a significant and independent and positive relationship between regulation of cognition and GPA, suggesting that students could plan, monitor and evaluate their learning processes. However, the relationship between the knowledge of cognition subscale and academic achievement is more complex. Correlational analysis showed a relationship between the variables. Nevertheless, the relationship was no longer significant in the regression analysis when knowledge of cognition was added up in the second step with regulation of cognition. The correlation may be attributed to the shared variance of knowledge of cognition with cognitive flexibility. Some possible explanations for this, as also noted by Costabile et al. (2013), are: (1) students develop effective regulatory behaviors through practice without being consciously aware of the strategies they use, (2) they prioritize subject content to meet immediate academic goals rather than focusing on the overall learning process, or (3) the transition from high school to university may have influenced their study techniques and academic performance.

### 7.1 Limitations

This study has several limitations. First, in line with the previous argument about the outcomes of inhibitory control, the potential reason to this lack of a role for inhibition in the current study is that the chosen task for inhibition, Go/No-Go Task included a low number of trials, thus reducing between subjects’ variability. The objective in the Go/No-Go Task is that the probability of participants responding falsely to no-go responses is increased when there are more ‘go’ responses than ‘no-go’ responses. In this study, the participants first performed a practice session and then started the actual task in the second block. The second block consisted of 25 go trials (to be responded to within 2,000 ms) and 5 no-go trials in total. As Young et al. (2018) pointed out, design variations such as go-to-no-go trial rate and task difficulty could influence the occurrence of false alarms, resulting in less sensitivity rates. Higher go rates, or easier trials may reduce the number of errors and thus impact the measurement of inhibitory control. With more trials and a shorter response time, inhibition could have been assessed with greater precision. Hence, the limited tasks and trial count and the distribution of go and no-go trials may have influenced the results on CC and affected the reliability of the inhibition measure. Friedman and Miyake (2017) argue that using multiple measures is necessary for accurate identification and understanding of these constructs. Overall, this has restricted the full exploration of the relationship of inhibitory control with academic achievement what can be considered a limitation of our study. Increasing the number of no-go trials and incorporating signal detection metrics in future studies could significantly improve the measurement precision of inhibitory control.

Secondly, It is important to highlight that metacognition is a broad and complex construct, which implies that its measurement with only one scale may not be sufficient to predict academic performance. The fact that only the self-report technique was used in the measurement of metacognition may also be a limitation. Many authors have raised concerns about the validity of using self-report questionnaires to assess metacognition and the need for improving these existing measures as it relies on self-report measures which introduce subjective bias into the results (Zepeda and Nokes-Malach, 2023; Double, 2025). Similarly, a study by Zepeda and Nokes-Malach (2023) which utilized a task-based metacognitive questionnaire including items adapted from the MAI, revealed that university students who actively monitored their comprehension during a learning activity tended to report less monitoring skill on the questionnaire. This finding indicates a potential bias in self-report assessment, which the authors suggested could stem from students being unaware of the extent of their struggles or underestimation of their competence. Similar results were also reported by highlighting unrealistic self-evaluations of students as measured by MAI (Hassan et al., 2022). Therefore, it is plausible that the data obtained through MAI in the present study may also reveal discrepancies between students’ actual monitoring processes and how they self-report.

Third, while not measured in the current study, internal and external factors influence metacognition, and were identified as highly significant (Bao et al., 2024). External factors such as social support, feedback and educational resources are crucial in shaping learners’ metacognitive abilities. Internal factors, including self-awareness, motivation, self-regulation and self-evaluation, and goal-directed behavior, also significantly contribute to academic success and metacognitive skills. The absence of an assessment of these factors, represents a limitation of the current study. As previously argued, motivation is an important variable in CC, significantly contributing to academic achievement (Deshmukh et al., 2025). It is also integrated as an affective component in an enriched model of metacognition proposed by Efklides (2014). Affective states such as confidence, frustration or motivation could influence MA and study behaviors which potentially explain the variability in GPA outcomes. Therefore, academic success is a multifaceted construct and influenced by a wide range of factors such as cognition, metacognition, motivation and other contextual factors beyond CC and metacognition (Molnár and Kocsis, 2024). Additionally, Pérez-González et al. (2022) emphasize the role of non-cognitive factors such as personality, and motivation in academic performance. Another relevant factor is different types of intelligence and their interaction with GPA scores as it also linked to metacognition, influencing academic achievement (Son and Hausman, 2024). A meta-analysis demonstrated that emotional intelligence is the third most important variable in predicting academic achievement after intelligence and non-cognitive factors such as conscientiousness. On the other hand, intelligence is also a construct distinct from CC. While CC involves specific EFs, intelligence refers to broader cognitive capabilities (Friedman and Miyake, 2017). Therefore, although both CC and metacognition are important, they are not the sole determinants of academic outcomes.

The fourth limitation relates to the sample composition and size. The sample was predominantly female (83.9%), which may limit the generalizability of the findings. However, it is important to emphasize that the proportion of females in the sample aligns with the gender distribution among psychology professionals in Spain, where 81.61% of registered psychologists in 2023 were women, according to data from the Spanish National Statistics Institute (Instituto Nacional de Estadística, 2024). Regarding the sample size, the current findings might be underpowered due to the limited sample of participants. Although *a priori* power analysis for a simple model suggests that about 90 participants, as in our study, are enough with an alpha of 0.05 and for a desired power o 0.80, to detect betas of 0.30 for paths a and b respectively, the sample for detecting full mediation effects is bit larger, around 113 participants (Kenny, 2017) and introducing another mediator, demands a larger sample size. Consequently, the absence of full mediation effects and the absence of partial effects of knowledge of cognition on academic achievement and the partial effects of cognitive flexibility on regulation of cognition should be taken with caution. In favor of our analysis, we should indicate that simulations have shown that other factors such as the variability of the predictor and the mediators, something that is quite evident in our measures of GPA, may increase power (Fritz et al., 2015).

Lastly, the cross-sectional design did not allow for conclusions regarding causality or the direction of the relationships between CC, metacognition and academic achievement. Given that the central research question explored whether metacognitive components fully or partially mediate the relationship between CC and GPA, the absence of temporal data limits the ability to make definitive claims about this mediation. Without longitudinal data, it remains unclear whether these cognitive and metacognitive processes contribute to academic achievement or whether academic experiences shape these abilities. Longitudinal studies are necessary to discover the stability of these relationships over time and to establish whether metacognition serves as a causal factor linking CC to academic outcomes.

### 7.2 Suggestion for future studies

Future research should expand its scope to include motivational and affective elements to gain more complete understanding of metacognitive processes. Similarly, future studies should also aim to use sensitive and more representative measures for inhibition to explore its relationship in this triangle. More so, although knowledge of cognition did not significantly predict academic achievement, regulation of cognition’s positive relationship with GPA suggests that students who are able to effectively manage and adjust their thinking processes perform better academically. These findings also point out the importance of incorporating metacognitive support practices in teaching, to encourage the development of metacognitive skills in students as they practice and reflect on their learning processes (Dennis and Somerville, 2022).

Furthermore, studies in the future should consider exploring the influence of culture in academic success. Inhibition is one example of how CC components may vary and affect academic performance across cultural contexts. In one culture, different CC components may predict academic achievement in particular subjects, while in a different one, distinct components may affect academic performance in the same subjects (Georgiou et al., 2020).

## 8 Conclusion

In accordance with the objectives of the study, the findings provide partial support for our hypothesis. Specifically, the results demonstrate that cognitive flexibility and the regulation of cognition are each independently associated with academic achievement. However, knowledge of cognition does not emerge as a significant predictor. This suggests that CC and metacognition contribute to academic performance in complementary yet distinct ways.

Overall, the present study contributes to the literature by demonstrating how cognitive flexibility and the regulation of cognition are factors that influence academic achievement, offering a comprehensive perspective on the mechanisms underlying academic outcomes. Interestingly, knowledge of cognition did not directly contribute to academic achievement once regulation of cognition was included in the equation. This finding indicates that awareness of learning strategies alone is inadequate for achieving academic success. Instead, the capacity to actively monitor and regulate one’s own learning emerges as a critical factor. Collectively, these findings emphasize that academic achievement is contingent not only on cognitive and metacognitive abilities, but also on the effective application of learning strategies.

## Data Availability

The datasets presented in this study are available in the Open Science Framework (OSF) repository at the following link: https://osf.io/jv67y/.

## Footnotes

^1^ https://www.psytoolkit.org/experiment-library/go-no-go.html

^2^ https://www.psytoolkit.org/experiment-library/wcst.html
